# Measurement properties of Chinese-version assessment tools of health-related quality of life in patients with stroke: a systematic review

**DOI:** 10.1007/s11136-025-04050-6

**Published:** 2025-09-25

**Authors:** Linfei Ding, Yunxun Chen, Yan Zhang, Xin Fu, Qin Liu, Xiaoyu Li

**Affiliations:** 1 Mianyang Central Hospital, School of Medicine, University of Electronic Science and Technology of China, 621000 Mianyang, China; 2 School of Medicine, University of Electronic Science and Technology of China, 610000 Chengdu, China; 3 Dazhou Vocational College of Chinese Medicine, 635000 Dazhou, China; 4 School of Nursing, North Sichuan Medical University, 637000 Nanchong, China

**Keywords:** Assessment instrument, COSMIN, Measurement properties, HRQOL, Stroke, Systematic review

## Abstract

**Purpose:**

To systematically evaluate the measurement properties of Chinese versions of HRQOL scales for stroke and provide evidence-based recommendations for clinical and research use.

**Methods:**

Ten databases (e.g., CNKI, VIP, PubMed, Embase) were searched from inception to September 2024. Studies evaluating the measurement properties of Chinese HRQOL scales for stroke patients were included. Two researchers independently screened, extracted data, and assessed measurement properties and methodological quality using COSMIN standards.

**Results:**

Thirty-seven studies were included, reporting on content validity, structural validity, internal consistency, reliability, hypotheses testing for construct validity, responsiveness, and measurement error. Two tools demonstrated sufficient content validity and at least low-quality evidence of sufficient internal consistency (Category A), while 17 were classified as Category B.

**Conclusions:**

Although diverse, the overall quality of Chinese versions of HRQOL scales is suboptimal, and further studies are needed. MHIEC-ST and SAQOL-39 g are recommended, with further measurement properties studies needed to refine and enhance these tools.

## Introduction

Stroke, a spectrum of neurofunctional disorders caused by cardiovascular and cerebrovascular diseases, includes both hemorrhagic and ischemic types [1]. Recent data from the Global Burden of Disease Study identify stroke as the second leading cause of death and the third leading cause of disability worldwide, with its burden escalating rapidly, particularly in low- and middle-income countries [2]. In China, stroke posed a significant public health challenge in 2019, accounting for 3.94 million new cases and 2.19 million deaths, highlighting the pressing need for effective prevention and control strategies [3]. In recent years, the number of stroke survivors has continued to grow as the healthcare system has made advances in the treatment and care during the acute phase of stroke. However, the sequelae due to neurological damage remain largely unavoidable, with most survivors experiencing reduced quality of life as a result of physical disabilities [4]. A review has shown that psychosocial factors among stroke patients are strongly associated with an increased risk of stroke occurrence [5]. Traditional physiological measures often fail to fully capture the multifaceted challenges faced by stroke patients in medical, familial, and social domains. By contrast, patient-reported health-related quality of life (HRQOL) effectively reflects the impact of disease and treatment on disability and daily functioning. Consequently, HRQOL has become an essential outcome indicator in clinical practice and research for evaluating rehabilitation effectiveness in stroke patients.

Health-related quality of life (HRQOL) refers to changes in an individual’s perceived well-being resulting from disease or treatment. It is a subjective construct encompassing multiple dimensions, including physical, psychological, and social functioning. All assessment instruments included in this study measured at least one dimension of HRQOL [6]. Instruments used to measure HRQOL typically take the form of patient-reported outcome measures (PROMs) designed for self-assessment [7]. HRQOL tools can be categorized into two main types: generic and disease-specific tools. Generic scales include the 36-Item Short Form Survey, the 12-Item Short Form Survey, and the WHO Quality of Life-BREF, etc. Disease-specific scales for stroke include the Stroke and Aphasia Quality of Life Scale, Stroke Impact Scale, and Stroke-Specific Quality of Life Scale, etc [8]. Generic instruments facilitate cross-disease HRQOL comparisons, while disease-specific tools are more sensitive to particular conditions, such as disease progression or the effects of clinical interventions [9]. A literature review indicates that no comprehensive systematic review has been conducted on the measurement properties of HRQOL tools specifically for stroke patients. The COSMIN (Consensus-based Standards for the Selection of Health Measurement Instruments) guidelines, which provide a comprehensive framework for assessing the psychometric properties of PROMs [10], offer a valuable set of standards for evaluating both measurement attributes and methodological quality. Guided by COSMIN, this study aims to systematically evaluate the psychometric properties of Chinese versions of HRQOL tools for stroke patients, employing the GRADE approach to offer graded recommendations. The findings are intended to assist clinicians and researchers in China in selecting appropriate tools for evaluating the HRQOL in stroke patients.

## Methods

### Data sources and search strategy

This study undertook a comprehensive literature search across multiple Chinese and English databases, including China National Knowledge Infrastructure (CNKI), Wanfang Data, VIP Database, Chinese Biomedical Literature Database (CBM), PubMed, Web of Science, Scopus, Embase, Cochrane Library, and CINAHL. The search covered the period from each database’s inception to July 2025. To enhance sensitivity and specificity, the search strategy combined subject headings with free-text terms. Furthermore, a snowballing technique was utilized to trace citations in relevant studies, ensuring no potentially eligible research was overlooked. This study strictly adheres to the PRISMA statement [11] and COSMIN guidelines [10]. The search strategy for this study on PubMed is presented in Appendix 1. This study is registered with the Prospero website under registration CRD42024609516.

### Inclusion and exclusion criteria

#### Inclusion criteria

(1) All participants were stroke patients; (2) The study’s objective is the development or cross-cultural adaptation of HRQOL tools for stroke patients; (3) The study evaluates at least one measurement property of HRQOL tools for stroke patients.

#### Exclusion criteria

(1) HRQOL tools that are not in Chinese; (2) Tools used exclusively as outcome measures without assessment of their measurement properties; (3) Tools employed solely for validating another measurement tool. (4) Review articles, systematic reviews, and other types of secondary literature; (5) Studies for which the full text is unavailable; (6) Duplicate publications, with preference given to the version containing more comprehensive data; (7) Studies focusing exclusively on a single domain of HRQOL, such as fatigue, depression.

### Study screening and data extraction

The processes of literature screening, data extraction, and quality assessment were independently conducted by two researchers in strict accordance with the COSMIN guidelines. Any disagreements during the evaluation process were resolved through discussion; if a consensus could not be reached, a third researcher was consulted to make the final decision. All participating researchers had completed coursework in evidence-based nursing and had thoroughly studied the latest version of the COSMIN guidelines, ensuring a rigorous and accurate evaluation process.

To enhance the efficiency and accuracy of literature management, EndNote 21.4 was employed for both automatic and manual deduplication of records. During the initial screening phase, review articles were excluded by applying document type filters. The titles and abstracts of the remaining articles were then reviewed to exclude those not meeting the study population or content criteria. Full-text reviews were conducted for the final selection to ensure alignment with the study’s inclusion criteria.

Data extraction included the following information: first author, year of publication, study location, instrument name, sample size, number of dimensions/items, retest interval, scoring method, completion time, and measurement properties.

### Quality appraisal

#### Methodological quality appraisal

The methodological quality of measurement properties in the included studies was evaluated using the COSMIN Risk of Bias Checklist. This checklist comprises 10 sections with a total of 116 items, covering methodological standards for scale development, content validity, internal consistency, structural validity, reliability, and other measurement properties. Each item is rated as “very good,” “adequate,” “doubtful,” “inadequate,” or “not applicable.” During the assessment, the “worst score counts” principle was applied, meaning that if any item within a measurement property was rated as “inadequate,” the overall methodological quality of that property was deemed “inadequate” [12].

*Measurement property appraisal*: (1) Content Validity: Content validity refers to the extent to which a scale’s items adequately reflect the construct it intends to measure [13], recognized as the most critical measurement property. COSMIN has developed a specific set of 10 criteria to evaluate content validity quality: five assess relevance, one evaluates comprehensiveness, and four assess comprehensibility [14]. This study included several generic health-related quality of life instruments (e.g., SF-36). As these instruments lack items that assess the impact of stroke-specific symptoms on patients’ quality of life, their comprehensiveness was rated as “insufficient” in this review. The assessment considered the results of PROMs development, the methodological quality of content validity study, and the reviewer’s rating, evaluating relevance, comprehensiveness, and comprehensibility separately. Each item can be rated as “sufficient”, “insufficient”, or “indeterminate”. The overall content validity for these three aspects was then summarized as “sufficient”, “Insufficient”, or “inconsistent”, with “indeterminate” results being rare due to the availability of reviewer opinions. The COSMIN guidelines recommend that content validity quality be assessed first; if strong evidence indicates insufficient content validity, further evaluation of other measurement properties can be omitted, with a final evidence recommendation provided. (2) Other Measurement Properties: Based on the criteria for good measurement properties [10], structural validity, internal consistency, reliability, cross-cultural validity/measurement invariance, and hypothesis testing for measurement properties are evaluated, with ratings of “sufficient,” “indeterminate,” or “insufficient.” The guidelines recommend assessing structural validity second, as it is a prerequisite for evaluating internal consistency. When structural validity is assessed using an exploratory factor analysis (EFA) design, a cumulative variance explained of over 50% or factor loadings of > 0.4 for dimensions are rated as “sufficient”; otherwise, they are rated as “insufficient” [15]. (3) Research hypothesis: The study team formulated the following hypotheses for hypothesis testing of construct validity and responsiveness: (a) Convergent Validity: Correlation coefficients should exceed 0.5 with tools measuring the same construct, range from 0.3 to 0.5 with related but different constructs, and fall below 0.3 with unrelated constructs. (b) Discriminant Validity: In the evaluation of the SF-36, SIS, SS-QOL, QOLISP, and DHI, the total quality-of-life scores and domain-specific scores differ by at least 10 points among general chronic disease patients and stroke patients, as well as between patients with severe and mild stroke, those who are dependent and independent in daily activities, and those with severe and mild disability. For the assessment of the Spitzer QLI, general chronic disease patients score 1.5 points higher than stroke patients, whereas cancer patients score 1.5 points lower than stroke patients. (c) Responsiveness: Following standardized interventions, stroke patients are expected to show substantial improvement in quality of life, with effect sizes for the overall scale and each dimension exceeding 0.8. Results consistent with these hypotheses in over 75% of cases were rated as sufficient; consistency below 75% is rated as insufficient.

#### Quality synthesis

First, it is necessary to determine whether the findings on each measurement property across studies are consistent. If results are consistent, they can be qualitatively summarized and compared to criteria for good measurement performance, with ratings of “sufficient” (+), “insufficient” (−), “inconsistent” (±), or “indeterminate” (?). If findings are inconsistent, several strategies may be employed: (a) investigate reasons for inconsistency and conduct subgroup analysis, (b) refrain from summarizing results and avoid rating the evidence, or (c) draw conclusions based on the majority of consistent findings, with potential downgrading due to inconsistency. When synthesizing inconsistent findings, greater emphasis is placed on high-quality and recent studies. Following the updated GRADE approach, the initial evidence quality rating is “high.” For content validity, downgrading may occur due to methodological bias, inconsistency, and indirectness; for other measurement properties, downgrading can occur due to methodological bias, inconsistency, indirectness, and imprecision. Evidence quality is ultimately rated as high, moderate, low, or very low.

#### Evidence level synthesis and recommendations

##### Recommendation

Measurement tools are categorized as “A” if they demonstrate “sufficient content validity at any quality level and at least low-quality evidence of sufficient internal consistency,” which can be recommended for use. Tools with high-quality evidence indicating insufficient measurement properties are categorized as “C,” which should not be recommended for use. Measurement tools not meeting the criteria for “A” or “C” are categorized as “B,” which have the potential to be recommended for use.

## Results

### Literature search

**Fig. 1:**
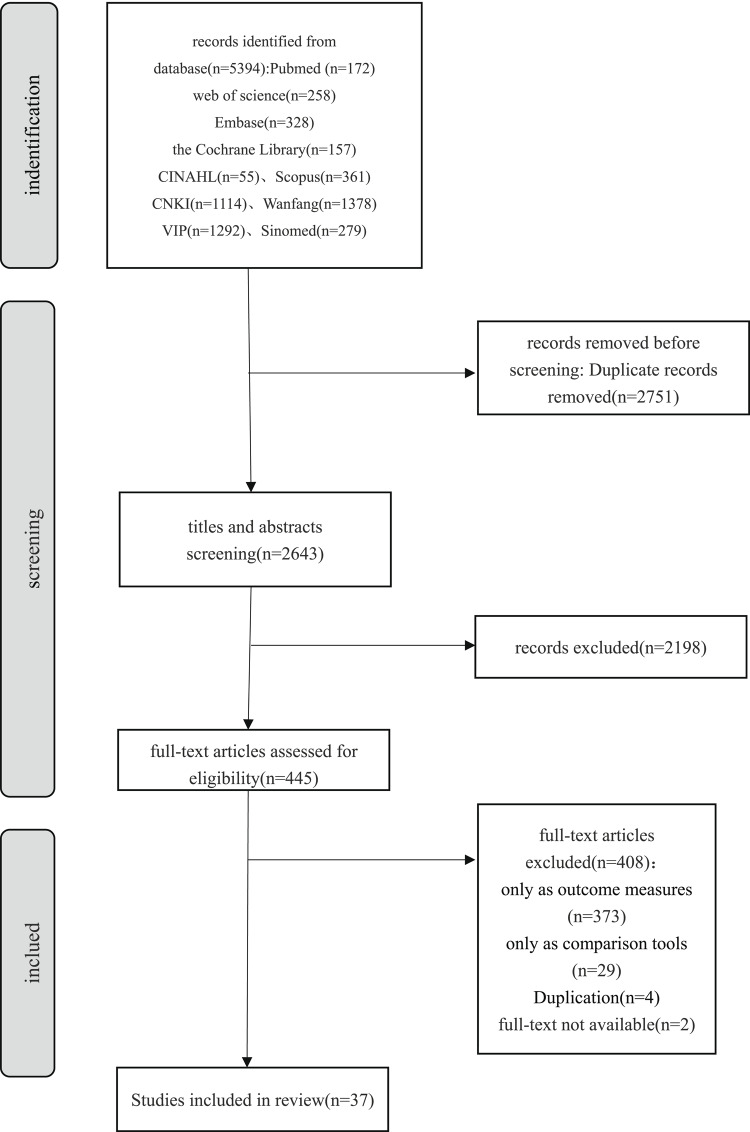
Literature screening process

In this study, we performed a comprehensive literature search across Chinese and English databases, yielding a total of 5394 articles—4063 from Chinese databases and 1331 from English databases. After automatic and manual deduplication, 2643 unique articles were retained for initial screening. During this stage, we first excluded non-relevant reference types, such as reviews, conference papers, and guidelines. Subsequently, we screened titles and abstracts to exclude studies that did not align with the target population or research focus. As a result, 445 articles were selected for full-text review. In this final stage, a total of 37 eligible articles [16–52] were identified, covering 21 PROMs and a total of 205 studies of measurement properties. Specifically, there were 22 studies on content validity (with each aspect evaluated separately), 26 on structural validity, 65 on internal consistency, 38 on reliability, 48 on hypotheses testing for construct validity, 5 on responsiveness, and 1 on measurement error. A detailed flowchart of the literature screening process is provided in Fig. 1.

### Study description

This study included 37 articles to evaluate the psychometric properties of HRQOL tools for stroke patients, encompassing 205 psychometric studies [16–52]. Among these, three articles [50–52] focused solely on item selection for content validity evaluation and are therefore not individually listed in the tables. Eleven articles [24, 25, 41–46, 50–52] developed original scales specifically for assessing HRQOL among Chinese stroke patients, while the remaining 26 focused on the psychometric properties of foreign scales adapted for use in China. Specifically, eight articles [16–23] analyzed generic HRQOL scales; three articles [47–49] evaluated scales for patients with dysphagia; and the remaining 26 articles addressed scales designed for patients with stroke. Regarding publication dates, 21 studies [18–20, 23, 26–30, 35–37, 41–46, 50, 52] were published before 2010, covering diverse locations across China, including Hong Kong, Macau, Taiwan, Tangshan, Tianjin, Zhengzhou, Sichuan, and Guangzhou. Additionally, one study [34] validated the Chinese version of the Stroke and Aphasia Quality of Life Scale in a Singaporean population. Most participants were stroke patients with mild to severe impairment who could cooperate during assessment, with a higher proportion of men than women. Sample sizes ranged from 30 to 800, but only nine studies reported the duration of illness. Regarding assessment methods, four articles employed proxy or professional evaluations, while the rest relied on patient self-reports. Only three studies reported ceiling/floor effects. Notably, none of the cross-culturally adapted scales described their scale development process. Over half (63%) of the studies did not assess content validity, although structural validity (80%) and internal consistency (93%) were frequently reported. None of the included studies evaluated cross-cultural validity/measurement invariance or criterion validity.

The HRQOL tools reviewed included a variety of instruments: World Health Organization Quality of Life-BREF (WHOQOL-BREF), Medical Outcomes Study Short Form-36 (SF-36), Medical Outcomes Study Short Form-12 (SF-12), Spitzer Quality of Life Index (QLI), Multidimensional Health Measurement Instruments System for Elderly Patients with Stroke (MHIEC-ST), Quality of Life Instrument for Patients with Stroke (QLICD-ST), Stroke Impact Scale (SIS), Stroke Impact Scale 3.0 for Proxy (proxy-SIS), Stroke and Aphasia Quality of Life Scale (SAQOL-39 g), The Stroke Specific Quality of Life Scale (SS-QOL), The Short Version of Stroke-specific Quality of Life (SV-SS-QOL), Quality of Life Inventory for Cerebral Apoplexy Patients (QOLI-CAP), Li et al.’s Traditional Chinese Medicine (TCM) Stroke Quality of Survival Scale, Cao et al.’s Quality of Life Scale for Stroke, He et al.’s Quality of Life Scale for Stroke, Quality of Life Instruments for Stroke Patients (QOLISP), the Eating Disorders Quality of Life (EDQOL), Dysphagia Handicap Index (DHI), and Swallowing Quality of Life Questionnaire (SWAL-QOL). Further details are provided in Table 1.

**Table 1:** Basic characteristics of the included literature

Author	Instrument	Site	Publication years	Study type	Response option	Mode of administration	Sample size	Age mean ± SD (range) year	Gender (% male)	Duration	Dimensions (No of items)	Retest intervals
Hou et al. [15]	WHOQOL-BREF	Tianjin	2013	Cross-sectional	1–5	Self-report	83	47–88	63/64%	–	4/26	–
Li et al. [16]	SF-36	Fujian, etc., multi-centers	2017	Cross-sectional/ cohort	–	Self-report	377	60.95 ± 9.89	244/65%	43 ± 19d	8/36	7d
Guo et al. [17]	SF-36	Guangzhou	2005	Cross-sectional	Tota l: 0–100	Self-report	197	66 ± 9.9	–	–	8/36	–
Zhang et al. [18]	SF-36	Sichuan	2001	Cross-sectional/ cohort	–	Self-report	150	42–82	92/61%	–	8/36	7d
Lotus et al. [19]	SF-36	Tanwan	2009	Cross-sectional	Total : 0–100	Self-report	98	73.1 ± 5.9	54/55.1%	–	8/36	–
Xu et al. [20]	SF-36	Xiamen	2023	Cross-sectional	Total : 0–100	Self-report	308	68.7 ± 12.1	166/54.07%	10.5 ± 6.77y	8/36	–
Liao et al. [21]	SF-12	Guangdong	2014	Cross-sectional/ cohort	Total : 0–100	Self-report	46	59.92 ± 12.23	28/61%	7.25 ± 1.35mon	2/12	7d
Gao et al. [22]	Spitzer QLI	Guangdong	1995	Cross-sectional	0–2	Professional rating	76	median 57	49/64%	–	5/5	–
Ding et al. [23]	MHIEC-ST	Shanghai, etc., multi-centers	2019	Cross-sectional/ cohort	1–5	Self-report	227	60–95	169/74%	–	Common modules 3/9 stroke-specific modules 3/7	–
Sun et al. [24]	QLICD-ST	Yunnan	2011	Cross-sectional/ cohort	1–5	Self-report	100	21–86	73/73%	–	Common modules 3/36 stroke-specific modules 1/18	2d
Lan et al. [25]	SIS	Guangzhou	2004	Cross-sectional/ cohort	1–5	Self-report	30	41–78	19/63%	–	8/59	24 h
Lan et al. [26]	SIS	Guangzhou	2005	Cross-sectional	1–5	Self-report	180	41–78	105/58%	–	8/59	–
Lan et al. [27]	SIS	Guangzhou	2006	Cross-sectional/ cohort	1–5	Self-report	180	41–78	105/58%	–	8/59	–
Zhu et al. [28]	SIS	Fujian	2006	Cross-sectional/ cohort	1–5	Self-report	130	47–83	74/56.92%	–	8/59	3d
Qi et al. [29]	Proxy -SIS	Guangzhou	2007	Cross-sectional	1–5	Proxy-report	117	–	–	–	8/59	–
Tian et al. [30]	SAQOL-39 g	Xuzhou	2020	Cross-sectional/ cohort	1–5	Self-report	88	55.27	64/73%	–	3/39	24 h
Guan et al. [31]	SAQOL-39 g	Guangzhou	2017	Cross-sectional/ cohort	1–5	Proxy-report	84	55.26 ± 15.65	68/81%	–	3/39	24 h
Lin et al. [32]	SAQOL-39 g	Fujian	2013	Cross-sectional/ cohort	1–5	Self-report	121	39–82	73/60%	–	3/39	2w
Guo et al. [33]	SAQOL-39 g	Singaporean	2016	Cross-sectional/ cohort	1–5	Self-report	58	–	–	–	3/39	7d
Wang et al. [34]	SS-QOL	Tangshan	2003	Cross-sectional	Total :0-100	Self-report	80	64.1(mean)	–	–	12/49	–
Li et al. [35]	SS-QOL	Handan	2007	Cross-sectional	Total :0-100	Self-report	60	62.7(mean)	–	–	12/49	–
Li et al. [36]	SS-QOL	Handan	2008	Cross-sectional	Total :0-100	Self-report	60	62.7	–	–	12/49	1w
Suzanne et al. [37]	SS-QOL	Hong Kong	2017	Cross-sectional/ cohort	1–5	Self-report	135	30–85	63.70%	6.08 ± 5.24y	11/47	4w
Tang et al. [38]	SV-SS-QOL	Shanxi、Hainan	2021	Cross-sectional	1–5	Self-report	156	42–83	86/55.13%	7-172d	2/12	2w
Ted et al. [39]	SV-SS-QOL	Hong Kong	2023	Cross-sectional	1–5	Self-report	184	23–64	113/61%	0.85-5.9y	1/12	2mon
Li et al. [40]	QOLI-CAP	Hunan	1997	Cross-sectional	1–5	Professional rating/ Self-report	89	35–85	61/69%	1-3650d	4/63	–
Li et al. [41]	TCM Stroke Quality of Survival Scale	Guangzhou	2008	Cross-sectional	–	Self-report	272	–	167/61.3%	–	4/60	–
Li et al. [42]	TCM Stroke Quality of Survival Scale	Guangzhou	2008	Cross-sectional/ cohort	–	Self-report	272	–	167/61.3%	–	4/60	3-10d
Cao et al. [43]	QOL Scale for Stroke	Shanghai, etc., multi-centers	2003	Cross-sectional/ cohort	–	Self-report	800	45–80	56.20%	0.66 ± 0.5y	5/22	24 h
He et al. [44]	QOL Scale for Stroke	Sichuan	1995	Cross-sectional/ cohort	1–6	Self-report	32	56 ± 5.2	18/56%	–	5/25	4-7d
Hu et al. [45]	QOLISP	Hunan	2006	Cross-sectional/ cohort	1–5	Self-report	120	–	–	–	4/36	24–48 h
Wang et al. [46]	EDQOL	Zhengzhou	2015	Cross-sectional/ cohort	1–5	Self-report	240	45–69	135/56%	–	5/25	2w
Xiang et al. [47]	DHI	Nanjing	2021	Cross-sectional/ cohort	0–4	Self-report	308	36–82	204/66%	–	3/25	2w
Tan et al. [48]	SWAL-QOL	Macau	2016	Cross-sectional/ cohort	1–5	Self-report	82	23–90	50/63%	10.12 ± 6.54y	2/44	24–48 h

Author	Instrument	Re-test sample	Time to complete (min)	Target population	Floor/ceiling effect	Recall period (week)	Measurement property indicators
Hou et al. [15]	WHOQOL-BREF	–	–	Outpatient clinic	–	2	EFA: CV = 74.73%
Li et al. [16]	SF-36	75	–	stroke with the first ever	–	4	EFA: CV = 67.3% Cronbach’s α = 0.318–0.958 ICC = 0.678–0.884
Guo et al. [17]	SF-36	–	–	hospital patients/ Outpatient clinic	–	4	EFA: CV = 77% Cronbach’s α = 0.56–0.95 Convergent validity →SF and BI: *r* = 0.821, MH and Spitzer QLI: *r* = 0.900 GH and Spitzer QLI: *r* = 0.455 Discriminant validity→ The difference of scores between severe and mild stroke was consistent with the hypothesis in 6 dimensions (+) and inconsistent in 2 dimensions (+). The difference in scores between dependent and independent patients was consistent with the hypothesis on all 8 dimensions (+).
Zhang et al. [18]	SF-36	20	–	Stroke with onset of 6–9 months	–	4	Cronbach’s α = 0.78-0.96 ICC = 0.64–0.95 Discriminant validity→ The difference in scores between severe and mild stroke was consistent with the hypothesis in 6 dimensions (+) and inconsistent in 2 dimensions (+).
Lotus et al. [19]	SF-36	–	–	Stroke without cognitive impairment	ceiling: RP/BP/SF/RE floor: RP/RE/SF/PF	4	Cronbach’s α = 0.71–0.95 Discriminant validity→ The difference in scores between dependent and independent patients was consistent with the hypothesis in 6 dimensions (+) and inconsistent in 2 dimensions (+).
Xu et al. [20]	SF-36	–	–	Community-dwelling stroke patients	Floor: PF/RF/RE/SF/ Ceiling: RE	–	EFA: CV = 69.9% Cronbach’s α = 0.648–0.985
Liao et al. [21]	SF-12	46	–	Patients with a first stroke more than 3 months ago	–	4	Total Cronbach’s α = 0.903 ICC0.726–0.912
Gao et al. [22]	Spitzer QLI	–	–	Stroke inpatients without coma or hemiparesis	–	–	EFA: CV = 100% Convergent validity → Spitzer QOLI and Fugl-Meyer, WIS, ADL: *r* = 0.38–0.64(*p* < 0.01) Discriminant validity→ The difference in scores between low back pain and stroke was consistent with the hypothesis (+).
Ding et al. [23]	MHIEC-ST	164	–	Stroke over 60 years old	–	–	Common modules: CV = 67.81% Stroke-specific modules: CV = 79.97 Common modules: Cronbach’s α = 0.79 Stroke-specific modules: Cronbach’s α = 0.83 ICC: *r* = 0.83–0.95
Sun et al. [24]	QLICD-ST	92	–	Stroke with some literacy skills	–	–	Common modules: CV = 68.394% Stroke-specific modules: CV = 71.483 Common modules: Cronbach’s α = 0.8729 Stroke-specific modules: Cronbach’s α = 0.8601 ICC = 0.809-0.865 Convergent validity→ QLICD-ST and SF-36: *r* = 0.27-0.73 Responsiveness→ post-intervention: 2 dimensions had large effect sizes that were consistent with the hypothesis (+) and 2 dimensions that were inconsistent with the hypothesis (-).
Lan et al. [25]	SIS	30	45 min–2 h	Stroke with a first onset of 1 month	–	1, 2, 4	Cronbach’s α = 0.621-0.923 ICC = 0.578–0.994
Lan et al. [26]	SIS	–	–	Stroke with a first onset of 1 month	–	1, 2, 4	EFA: CV = 62.341% Convergent validity→ SIS and FCA, SF-36: *r* = 0.27–0.87
Lan et al. [27]	SIS	–	–	Stroke with a first onset of 1 month	–	1, 2, 4	Responsiveness→ post-intervention: 6 dimensions had large effect sizes that were consistent with the hypothesis (+) and 2 dimensions that were inconsistent with the hypothesis (-). Discriminant validity→ The difference in scores between stroke and chronic disease patients in general was consistent with the hypothesis in 6 dimensions (+) and inconsistent in 2 dimensions (+).
Zhu et al. [28]	SIS	20	20–30	stroke with the first ever	–	–	EFA: CV = 81.13% Cronbach’s α = 0.8108–0.9663 ICC = 0.7289–0.9650
Qi et al. [29]	proxy -SIS	–	15–20	Stroke over 18 years old.	–	1, 2, 4	Cronbach’s α>0.8 Convergent validity→ proxy -SIS and SF-36, BI, Hamilton: *r* = 0.515-0.872 Discriminant validity→ The difference of scores between mild and severe disabilities was consistent with the hypothesis on all 8 dimensions (+).
Tian et al. [30]	SAQOL-39 g	88	21.61 ± 4.38	Patients with aphasia in their first stroke	–	–	EFA: Factor loadings > 0.4 for each dimension Cronbach’s α = 0.881–0.946 ICC = 0.803–0.973
Guan et al. [31]	SAQOL-39 g	60	21.4 ± 4.37	Patients with aphasia in their first stroke	–	–	EFA: CV = 59.7% Cronbach’s α = 0.882–0.947 ICC = 0.804–0.974
Lin et al. [32]	SAQOL-39 g	20	15	Patients with aphasia in their first stroke	–	–	EFA: CV = 54%, Cronbach’s α = 0.89–0.95 ICC = 0.91–0.97
Guo et al. [33]	SAQOL-39 g	28	–	Patients with a first-ever stroke	–	–	Cronbach’s α = 0.94–0.97 ICC = 0.92-0.99 Convergent validity →SAQOL-39 g and BI, MMSE et al.: *r* = 0.28–0.84
Wang et al. [34]	SS-QOL	60	15	Patients with a mild to moderate stroke onset for 1 month	–	–	Cronbach’s α = 0.82–0.98 Discriminant validity→ The difference of scores between mild and severe disabilities was consistent with the hypothesis in 9 dimensions (+) and inconsistent in 3 dimensions (+) Convergent validity →SS-QOL and SF-36, NIHSS, BI, Zung, MMSE: *r* = 0.10–0.84
Li et al. [35]	SS-QOL	–	–	Patients with a mild to moderate stroke onset for 3 months	–	–	Convergent validity→ SS-QOL and SF-36, NIHSS, BI, Zung, MMSE: *r* = 0.08–0.89 Discriminant validity→ The difference in scores between mild and severe disabilities was consistent with the hypothesis on all 12 dimensions (+)
Li et al. [36]	SS-QOL	60	–	Stroke within 24 h of onset	–	–	Cronbach’s α = 0.82–0.98
Suzanne et al. [37]	SS-QOL	35	45	Stroke who speaks Cantonese	Floor: no ceiling: language / vision / Basic needs/Transfer	–	EFA: CV = 67.25% Cronbach’s α = 0.63-0.93 ICC = 0.57 Convergent validity→ SS-QOL and SF-36: *r* = 0.2–0.68, SS-QOL and SSEQ-c, FAI, BI༚*r* = 0.43–0.68
Tang et al. [38]	SV-SS-QOL	50	–	Stroke recovery patients	–	–	CFA: CFI = 0.91, RMSEA = 0.062 Cronbach’s α = 0.832-0.891 ICC = 0.818–0.877
Ted et al. [39]	SV-SS-QOL	–	15	Stroke in inpatient or community rehabilitation centers	–	–	CFA: CFI = 0.951 Mcdonald’s Ω = 0.853 SEM = 3.68, SDC = 7.22 Convergent validity → SV-SS-QOL and SHS, RSES, SF-12: *r* = 0.41–0.60, SV-SS-QOL and MRS, HADS: *r* = − 0.38–0.61
Li et al. [40]	QOLI-CAP	–	–	Stroke inpatients	–	–	EFA: CV = 67.8% Cronbach’s α = 0.7963
Li et al. [41]	TCM Stroke Quality of Survival Scale	–	–	Strokes who can express their views clearly	–	–	EFA: CV = 59.087%
Li et al. [42]	TCM Stroke Quality of Survival Scale	33	23.29 ± 7.66	Strokes who can express their views clearly	–	–	Cronbach’s α > 0.8 ICC = 0.9047–0.9675 Convergent validity→ TCM Stroke Quality of Survival Scale and SS-QOL: *r* = 0.711
Cao et al. [43]	QOL Scale for Stroke	120	12.26 ± 3.31	Patients with mild, moderate, or severe stroke	–	–	EFA: CV = 60.27% Cronbach’s α = 0.65–0.76 ICC = 0.882-1 Convergent validity → QOL Scale for Stroke and NHP: *r* = 0.450–0.604
He et al. [44]	QOL Scale for Stroke	32	20–30	Stroke patients	–	–	EFA: CV = 82.6 ICC = 0.96
Hu et al. [45]	QOLISP	120	13.98 ± 3.87	Strokes that are lucid	–	1	EFA: CV = 62.45% Cronbach’s α = 0.78-0.94 ICC = 0.89-0.97 Convergent validity →QOLISP and WHOQOL-BREEF, SF-36: *r* = 0.59, 0.45 Discriminant validity→ Differences in scores between healthy individuals and stroke patients on 4 dimensions were consistent with the hypothesis (+), and 1 was inconsistent (−).
Wang et al. [46]	EDQOL	30	–	Patients with dysphagia after their first stroke	–	–	EFA: CV = 61.299% Cronbach’s α = 0.839-0.932 ICC = 0.751–0.874 Convergent validity→ EDQOL and SF-36: *r* = − 0.718
Xiang et al. [47]	DHI	30	12	Stroke with dysphagia	–	–	EFA: CV = 68.77% Cronbach’s α = 0.8–0.96 ICC = 0.80-0.88 Discriminant validity → Differences in scores between mild dysphagia and severe dysphagia were consistent with the hypothesis on all 3 dimensions (+). Convergent validity → DHI and SWAL-QOL: *r* = 0.725
Tan et al. [48]	SWAL-QOL	30	–	Stroke with dysphagia	–	–	EFA: CV = 57.438% ICC = 0.908-0.975 Cronbach’s α = 0.769–0.973

### Methodological quality and measurement property assessment

Lan et al. reported multiple psychometric properties of the SIS across three articles, all based on the same dataset. Similarly, Li et al. published two articles on various psychometric properties of the SS-QOL, and Li et al. reported multiple psychometric properties of the TCM Stroke Quality of Survival Scale across two articles. For clarity, the findings on the psychometric properties of these scales have been consolidated for reporting purposes. As the SF-36, MHIEC-ST, and QLICD-ST are multidimensional instruments, the measurement properties of each subscale were assessed individually, and separate recommendations were formulated accordingly. Details are presented in Tables 2 and 3.

**Table 2:** Results of methodological quality and measurement properties of the included tools

Author	Instrument	Content validity				Structural validity	Internal consistency	Reliability	Hypothesis testing		Responsiveness	Measurement error
		MQ	Relevance	Comprehensiveness	Comprehensibility				Convergent validity	Discriminant validity		
Hou et al. [15]	WHOQOL-BREF	–	＋	−	＋	I/＋	D/＋	–	–	–	–	–
Li et al. [16]	SF-36	D	＋	−	＋	A/＋	–	–	–	–	–	–
Guo et al. [17]	SF-36	–	＋	−	＋	A/+	–	–	–	–	–	–
Zhang et al. [18]	SF-36	–	＋	−	＋	–	–	–	–	–	–	–
Lotus et al. [19]	SF-36	–	＋	−	＋	–	–	–	–	–	–	–
Xu et al. [20]	SF-36	–	＋	−	＋	A/+	–	–	–	–	–	–
Liao et al. [21]	SF-12	–	＋	−	＋	–	I/?	D/＋	–	–	–	–
Gao et al. [22]	Spitzer QLI	–	＋	−	＋	A/＋	–	–	V/+	V/+	–	–
Ding et al. [23]	MHIEC-ST	D	＋	＋	＋	A/＋	V/＋	D/＋	–	–	–	–
Sun et al. [24]	QLICD-ST	D	＋	＋	＋	I/＋	V/＋	I/＋	A/+	−	V/−	–
Lan et al. [25]	SIS	–	＋	＋	＋	I/＋	V/−	I/＋	A/+	V/+	V/+	–
Zhu et al. [28]	SIS	–	＋	＋	＋	I/＋	V/+	I/＋	–	–	–	–
Qi et al. [29]	Proxy -SIS	–	＋	＋	＋	–	V/?	–	A/+	V/+	–	–
Tian et al. [30]	SAQOL-39 g	–	＋	＋	＋	I ＋	V/+	I/＋	–	–	–	–
Guan et al. [31]	SAQOL-39 g	–	＋	＋	＋	I/＋	V/+	I/＋	–	–	–	–
Lin et al. [32]	SAQOL-39 g	D	＋	＋	＋	I/＋	V/+	A/＋	–	–	–	–
Guo et al. [33]	SAQOL-39 g	–	＋	＋	＋	–	V/+	D/＋	A/+	–	–	–
Wang et al. [34]	SS-QOL	–	＋	＋	＋	I/?	V/?	I/＋	A/+	V/+	–	–
Li et al. [36]	SS-QOL	–	＋	＋	＋	–	V/?	I/＋	A/+	V/+	–	–
Suzanne et al. [37]	SS-QOL	D	＋	＋	＋	I/＋	V/?	D/−	A/+	–	–	–
Tang et al. [38]	SV-SS-QOL	D	＋	＋	＋	V/−	V/＋	A/＋	–	–	–	–
Ted et al. [39]	SV-SS-QOL	–	＋	＋	＋	V/＋	V/?	/	A/+	–	–	A/＋
Li et al. [40]	QOLI-CAP	D	＋	＋	＋	I/＋	I/?	–	–	–	–	–
Li et al. [42]	TCM Stroke Quality of Survival Scale	D	＋	＋	＋	I/＋	V/＋	D/＋	A/+	–	I/＋	–
Cao et al. [43]	QOL Scale for Stroke	–	＋	＋	＋	A/＋	V/−	I/＋	A/+	–	–	–
He et al. [44]	QOL Scale for Stroke	–	＋	−	＋	I/＋	/	D/＋	–	D/＋	–	–
Hu et al. [45]	QOLISP	D	＋	＋	＋	I/＋	V/＋	I ＋	A/+	–	I/＋	–
Wang et al. [46]	EDQOL	D	＋	−	＋	A/＋	V/＋	A/＋	V/+	–	–	–
Xiang et al. [47]	DHI	D	＋	−	＋	A/＋	V/＋	A/＋	A/＋	–	–	–
Tan et al. [48]	SWAL-QOL	–	＋	＋	＋	I/＋	V/?	I/＋	–	–	–	–

Methodological quality / measurement property; MQ methodological quality; “+” sufficient, “–” insufficient, “?” indeterminate; “V” Very good, “A” adequate, “D” Doubtful, “I” inadequate

**Table 3:** Results of methodological quality and measurement properties of SF-36

Author	Instrument	Internal consistency								Reliability								Convergent validity			Discriminant validity							
		GH	PF	RP	RE	SF	BP	VT	MH	GH	PF	RP	RE	SF	BP	VT	MH	GH	PF	MH	GH	PF	RP	RE	SF	BP	VT	MH
Li et al. [6]	SF-36	V/−	V/+	V/+	V/+	V/−	V/+	V/−	V/+	D/−	D/+	D/+	D/+	D/−	D/−	D/−	D/+	–	–	–	–	–	–	–	–	–	–	–
Guo et al. [17]	SF−36	V/+	V/+	V/+	V/+	V/+	V/−	V/−	V/+	–	–	–	–	–	–	–	–	A/+	A/+	A/+	V/+	V/+	V/+	V/+	V/+	V/+	V/+	V/+
Zhang et al. [18]	SF−36	V/+	V/+	V/+	V/+	V/+	V/+	V/+	V/+	D/+	D/+	D/+	D/−	D/+	D/+	D/+	D/+	–	–	–	V/+	V/+	V/+	V/+	V/+	V/−	V/+	V/−
Lotus et al. [19]	SF−36	V/+	V/+	V/+	V/+	V/+	V/+	V/+	V/+	–	–	–	–	–	–	–	–	–	–	–	V/−	V/+	V/+	V/+	V/+	V/+	V/−	V/+
Xu et al.[20]	SF−36	V/+	V/+	V/+	V/+	V/−	V/−	V/+	V/+	–	–	–	–	–	–	–	–	–	–	–	–	–	–	–	–	–	–	–

Methodological Quality / Measurement Property; PF physical functioning; RP role limitation due to physical problems; BP bodily pain; GH general health; VT vitality; SF social functioning; RE role limitation due to emotional problems; MH mental health;

#### Content validity

Evaluating content validity requires consideration of the results of PROMs development, the methodological quality of content validity study, and the reviewer’s rating [14]. In this study, only five studies [41, 42, 46, 51, 52] described the development process of the PROMs, however, the methodological quality of these studies was rated as “inadequate” or “doubtful.” The remaining studies did not report any development process of the instruments specifically for stroke patients, and thus were rated as having “inadequate” methodological quality. Eleven articles [17, 24, 25, 33, 38, 39, 41, 42, 46–48], covering 22 studies in total, addressed at least one aspect of content validity, including relevance, comprehensiveness, and comprehensibility. Based on the reviewer’s rating, the remaining 74 studies for the unreported aspects of content validity received a quality rating. For studies employing quantitative designs, several [33, 38, 39, 41, 42, 46, 47] had sample sizes of patients and/or professionals that did not meet guideline recommendations, lacked details on the number of field experts or patients involved [25], or included experts from a limited range of disciplines [17]. Two studies [39, 48] did not specify whether they used quantitative or qualitative methods. For studies using qualitative designs, two [42, 47] failed to report whether interviewers were experienced or uniformly trained and omitted detailed descriptions of their data analysis methods. These limitations led to a “doubtful” rating for methodological quality. According to the 10 criteria for good content validity, 22 studies on content validity were rated as “sufficient”. Additionally, our research team evaluated whether each scale comprehensively covered all relevant domains of stroke patients’ quality of life, item wording was deemed reasonable and easily understandable. Based on these criteria, 74 content validity studies were rated as either “sufficient” or “insufficient”. Regarding the SF-36, it is a generic instrument that was not originally developed for stroke populations. Consequently, it lacks items that specifically address the impact of stroke-related symptoms on quality of life. Its content validity was therefore rated as “insufficient” in terms of comprehensiveness, both at the total scale and subscale levels. In contrast, the “relevance” and “comprehensibility” aspects were rated as “sufficient.” To avoid redundancy and given the consistency in rationale across subscales, we did not report separate content validity ratings for each subscale.

#### Structural validity

A total of 24 studies [16–18, 21, 23–25, 27, 29, 31–33, 35, 38–42, 44–49] reported on structural validity. Among these, two studies [39, 40] employed confirmatory factor analysis (CFA) and were rated as “very good” in methodological quality, while one study [35] applied cluster analysis and received a rating of “inadequate.” The remaining 21 studies conducted exploratory factor analysis (EFA). Due to insufficient sample sizes (fewer than five times the number of items), the methodological quality of 13 studies [16, 25, 27, 29, 31–33, 38, 41, 42, 45, 46, 49] was downgraded to “inadequate,” whereas the quality of the other studies was rated as “adequate.” Among studies using EFA, one [35] did not report the cumulative variance explained, resulting in an “indeterminate” rating for structural validity. The remaining studies demonstrated cumulative variances explained of over 50% or factor loadings > 0.4 for each dimension, thereby qualifying their structural validity as “sufficient”, Regarding CFA, one study [39] reported a CFI of 0.91 and an RMSEA of 0.062, which did not meet the standards for good measurement properties (CFI > 0.95 or RMSEA < 0.06), resulting in a rating of “insufficient” for structural validity. In contrast, one study [40] reported a CFI exceeding 0.95, meeting guideline standards and earning a “sufficient” rating for structural validity.

#### Internal consistency

According to the guidelines, the prerequisite for evaluating the internal consistency quality is that “there must be at least low-quality evidence supporting sufficient structural validity”. In this study, 28 articles [16–22, 24–26, 29–35, 37–41, 43, 44, 46–49]reported internal consistency. Study [16] only described item-to-dimension correlations without a comprehensive internal consistency analysis, leading to a “doubtful” rating for methodological quality. Studies [22, 41] reported internal consistency solely for the overall scale or a single dimension, omitting calculations for each dimension, and were therefore rated as “inadequate.” The remaining studies calculated Cronbach’s alpha for each dimension and were rated as “very good” in methodological quality. However, Studies [16, 22, 25, 30, 35, 37–41, 43, 46, 49] did not meet the requirement of “at least low-quality evidence of sufficient structural validity,” resulting in an “indeterminate” rating for internal consistency. Studies [24, 26, 44]reported Cronbach’s alpha values below 0.7 for certain subdimensions, resulting in an “insufficient” rating. Conversely, studies [29, 31–34, 47, 48] reported Cronbach’s alpha values exceeded 0.7 across all dimensions or subscales, earning a “sufficient” rating for internal consistency quality. The quality of internal consistency for each subscale of the SF-36 is presented in Table 3.

#### Reliability

A total of 22 articles reported test-retest reliability. Among them, the methodological quality of studies [22, 24, 34, 38, 43, 45] was rated as “doubtful”, while studies [25, 26, 29, 31, 32, 35, 37, 44, 46, 49] were rated as “inadequate”, Studies [35, 37] did not report Intraclass Correlation Coefficient (ICC) values, resulting in a reliability quality rating of “indeterminate.” In study [38], the ICC value was 0.57, which fell below the threshold of 0.7, leading to a reliability quality rating of “insufficient.” The remaining studies reported ICC values exceeding 0.7, achieving a reliability quality rating of “sufficient.” The quality of reliability for each subscale of the SF-36 is presented in Table 3.

#### Measurement error

Measurement error was reported in only one study [39], which was rated “adequate” in methodological quality and rated as “indeterminate” in measurement property quality.

### Hypotheses testing for construct validity

Fourteen studies [23, 25, 27, 30, 34, 35, 37, 38, 40, 42, 44, 46–48] assessed convergent validity. The methodological quality of studies [25, 27, 30, 34, 35, 37, 38, 40, 42, 44, 46, 48] was rated as “adequate,” while studies [23, 47] were rated as “very good.” findings indicated that over 75% of the correlation coefficients between the scales and comparison tools met the hypothesis, thus achieving a convergent validity quality rating of “sufficient.” Additionally, six studies [23, 27, 30, 35, 37, 45] reported discriminant validity. Study [45] lacked a detailed description of control group characteristics, earning a methodological quality rating of “doubtful” and a measurement property quality rating of “indeterminate”, The remaining studies provided clear descriptions of the differences between comparison groups and exhibited no statistical deficiencies, earning a methodological quality rating of “very good.” Over 75% of the results supported the study hypothesis, earning a known-groups validity quality rating of “sufficient.” The quality of construct validity for each subscale of the SF-36 is presented in Table 3.

### Responsiveness

Four studies [25, 28, 43, 46] reported on responsiveness. Studies [43, 46] failed to provide mean values or baseline standard deviations for pre- and post-intervention measurements, making effect size calculations impossible. Consequently, their methodological quality was rated as “inadequate”, and the measurement property quality was rated as “indeterminate.” In study [28], 78% (7/9) of the results aligned with the hypothesis, yielding a measurement property quality rating of “sufficient.” In contrast, study [25] reported that only 50% of the results for the QLICD-ST(CGD) were in line with the hypotheses, resulting in a rating of “insufficient.” Meanwhile, the results for the QLICD-ST(SPD) were fully consistent with the hypotheses and therefore received a rating of “sufficient.”

### Evaluation of measurement properties

(1) Content Validity: nine scales were rated as “inconsistent”, while 12 scales were rated as “sufficient.” (2) Structural Validity: One scale was rated as “inconsistent,” while 18 scales were rated as “sufficient.” (3) Internal Consistency: Four scales were rated as “sufficient,” three as “insufficient,” one as “inconsistent,” and ten as “indeterminate”. Internal consistency for each subscale of the SF-36 was rated as “sufficient.” (4) Reliability: 15 scales were rated as “sufficient,” with one scale rated as “insufficient.” The GH, RE, SF, BP, and VT dimensions of the SF-36 demonstrated “inconsistent” reliability, while the PF, RP, and MH dimensions showed “sufficient” reliability. (5) Hypothesis Testing: 13 scales were rated as “sufficient,” with one rated as “indeterminate”, construct validity for each subscale of the SF-36 is “sufficient.” (6) Responsiveness: two scale was rated as “sufficient,” one as “insufficient,” and two as “indeterminate.” (7) Measurement Error: Only one scale reported measurement error, with a quality rating of “indeterminate.”

### Grading the quality of evidence

Since all participants in this study were stroke patients, measurement properties were not downgraded due to “indirectness.” (1) Content Validity: Following guidelines, aspects of content validity not addressed in studies were evaluated based on reviewers’ opinions, with a quality rating of “very low.” Tools howed “inconsistent” content validity were not rated. MHIEC-ST(SPD), QLICD-ST(SPD), QOLISP, and the TCM Stroke Quality of Survival Scale were downgraded by two level due to risk of bias, resulting in a “low” quality rating. (2) Structural Validity: Eight scales were downgraded by three levels for risk of bias, resulting in a final quality rating of “very low.” two scales were downgraded by two levels to “low,” seven scales by one level to “moderate.” The SV-SS-QOL scale showed “inconsistent” results and could not be rated. (3) Internal Consistency: According to guidelines, the quality of evidence for structural validity serves as the baseline for internal consistency, with further downgrades applied for risk of bias, inconsistency, imprecision, or indirectness as needed. Eleven scales had “indeterminate” or “inconsistent” results and could not be rated. six scales were rated as “moderate,” one as “low”. Each subscale of the SF-36 was rated “high.” (4) Reliability: Seven scales were downgraded by two levels due to risk of bias, five by three levels, and four by one level. Additionally, seven scales were downgraded by two levels for imprecision, and four scales by one level. Ultimately, one scale was rated as “moderate,” three as “low,” and twelve as “very low.” The PF, RP, and MH dimensions of the SF-36 were downgraded by one level due to risk of bias, resulting in a “moderate” quality rating. (5) Hypothesis Testing: One study had “indeterminate” results and could not be rated. eight scales were downgraded by one level for risk of bias, and two by one level for imprecision. Overall, four scales achieved a “high” quality rating, eight were rated as “moderate,” and one as “low.” Each subscale of the SF-36 was rated as “high.” (6). Responsiveness: Two scales had “indeterminate” results and could not be rated. two scales were downgraded by two levels for risk of bias, resulting in a “low” quality rating. (7) Measurement Error: Only one study reported “indeterminate” results, and thus no quality rating could be assigned.

### Recommendations

The QLICD-ST(SPD) and SAQOL-39 g, categorized as “A”, are recommended for use, as they demonstrated “sufficient content validity with at least low-quality evidence supporting adequate internal consistency.” The remaining 19 scales did not meet the criteria for either A or C and were thus categorized as “B”. Detailed descriptions are provided in Tables 4 and 5.

**Table 4:** Summary of quality and recommendations for the included tools

Author	Instrument	Content validity		Structural validity		Internal consistency		Reliability		Hypothesis testing		Responsiveness		Measurement error		Recommendations
		Summary of results	Rating	Summary of results	Rating	Summary of results	Rating	Summary of Results	Rating	Summary of results	rating	Summary of results	Rating	Summary of results	Rating	
Hou et al. [15]	WHOQOL-BREF	±	–	＋	VL	?	–	–	–	–	–	–	–	–	–	B
Li et al. [16]	SF-36	±	–	＋	H	–	–	–	–	–	–	–	–	–	–	–
Guo et al. ^[17]^																
Zhang et al. [18]																
Lotus et al. ^[19]^																
Xu et al. [20]																
Liao et al. [21]	SF-12	±	–	–	–	?	–	＋	VL	–	–	–	–	–	–	B
Gao et al. [22]	Spitzer QLI	±	–	＋	M	–	–	–	–	＋	H	–	–	–	–	B
Ding et al. [23]	MHIEC-ST	＋	VL	＋	M	＋	M	＋	L	–	–	–	–	–	–	A
Sun et al. [24]	QLICD-ST	＋	VL	＋	VL	?	–	＋	VL	＋	L	−	M	–	–	B
Lan et al. [25]	SIS	＋	VL	＋	L	±	–	＋	VL	＋	H	＋	H	–	–	B
Zhu et al. [28]																
Qi et al. [29]	Proxy-SIS	＋	VL	–	–	−	–	–	–	＋	H	–	–	–	–	B
Tian et al. [30]	SAQOL-39 g	＋	VL	＋	L	＋	L	＋	M	–	–	–	–	–	–	A
Guan et al. [31]																
Lin et al. [32]																
Guo et al. ^[33]^																
Wang et al. [34]	SS-QOL	＋	VL	＋	VL	?	–	−	VL	＋	H	–	–	–	–	B
Li et al. [36]																
Suzanne et al. [37]																
Tang et al. [38]	SV-SS-QOL	＋	VL	±	–	?	–	＋	L	＋	M	–	–	＋	–	B
Ted et al. [39]																
Li et al. [40]	QOLI-CAP	＋	VL	＋	VL	−	–	–	–	–	–	–	–	–	–	B
Li et al. [42]	TCM Stroke quality of survival scale	＋	M	＋	VL	?	–	＋	VL	＋	M	＋	–	–	–	B
Cao et al. [43]	QOL scale for stroke	＋	VL	＋	M	−	M	＋	VL	＋	L	–	–	–	–	B
He et al. [44]	QOL scale for stroke	±	–	＋	VL	−	–	＋	VL	＋	–	–	–	–	–	B
Hu et al. [45]	QOLISP	＋	M	＋	VL	?	–	＋	VL	＋	M	＋	–	–	–	B
Wang et al. [46]	EDQOL	±	–	＋	M	＋	M	＋	VL	＋	H	–	–	–	–	B
Xiang et al. [47]	DHI	±	–	＋	M	＋	M	＋	VL	＋	M	–	–	–	–	B
Tan et al. [48]	SWAL-QOL	＋	VL	＋	VL	?	–	＋	VL	–	–	–	–	–	–	B

+, sufficient; ?, indeterminate; −, insufficient; ±, inconsistent; H, high; M Moderate; L, low; VL, very low

**Table 5:** Summary of quality and recommendations for each subscale of the SF-36

Instrument	Subscale	Content validity		Internal consistency		Reliability		Hypothesis testing		
		Summary of Results	Rating	Summary of results	Rating	Summary of results	Rating	Summary of results	Rating	Recommendations
SF-36	GH	±	–	+	H	±	–	+	H	B
	PF	±	–	+	H	+	M	+	H	B
	RP	±	–	+	H	+	M	+	H	B
	RE	±	–	+	H	±	–	+	H	B
	SF	±	–	+	H	±	–	+	H	B
	BP	±	–	+	H	±	–	+	H	B
	VT	±	–	+	H	±	–	+	H	B
	MH	±	–	+	H	+	M	+	H	B

PF physical functioning; RP role limitation due to physical problems; BP bodily pain; GH general health; VT vitality; SF social functioning;

RE role limitation due to emotional problems; MH mental health

## Discussion

This systematic review evaluated 21 tools used to assess HRQOL in stroke patients. Two tools met the criteria for A-level classification: QLICD-ST(SPD) and SAQOL-39 g. General quality of life scales and dysphagia-specific scales showed limitations in content validity for stroke patients; therefore, researchers and clinicians should select appropriate scales tailored to specific research or clinical needs. The following presents the evaluation results and recommendations from our research team regarding measurement property studies in the literature, based on COSMIN guidelines.

### Content validity

Content validity refers to the degree to which a scale adequately represents the construct it is intended to measure, making it a critical factor when selecting measurement tools [13]. With the advancement of general HRQOL scales and disease-specific scales for stroke patients, precision in measuring constructs has improved. However, this progress has also introduced heterogeneity in clinical research outcomes. To address this, the World Health Organization (WHO) has recommended that HRQOL measurement tools cover three core dimensions: physical, psychological, and social functioning. For disease-specific assessments, an additional symptom dimension is also advised [53]. General HRQOL scales such as the WHOQOL-BREF, SF-36, SF-12, and the Spitzer Quality of Life Index do not incorporate a symptom dimension. Consequently, their content validity was rated as “insufficient” in terms of comprehensiveness by our research team. Similarly, tools like the EDQOL, DHI, and He et al.’s QOL Scale for Stroke lack a symptom and/or physical dimension, resulting in “insufficient” ratings for comprehensiveness. In contrast, disease-specific tools such as MHIEC-ST(SPD), QLICD-ST(SPD), SIS, SAQOL-39 g, SS-QOL, SV-SS-QOL, QOLI-CAP, the TCM Stroke Quality of Survival Scale, Cao et al.’s QOL Scale for Stroke, and QOLISP encompass multiple dimensions of stroke patients’ HRQOL, with clear and comprehensible items. These scales received a “sufficient” rating for content validity. Researchers and clinicians can select appropriate HRQOL measurement tools based on their needs. This paper also highlights common issues in the scale development or localization process, such as the insufficient number of professionals and or patients meeting the recommended standards for qualitative interviews or quantitative surveys. Additionally, it was unclear whether interview guides or topics were provided during qualitative interviews, and there was a lack of detailed data coding and analysis procedures. Therefore, it is recommended that future scale development follow strict qualitative research design requirements, conducting interviews with both patients and professionals to enhance the content validity of the tools. For the cross-cultural adaptation of foreign scales, the standard process of translation, back-translation, cultural adaptation, and pre-testing should be followed [54], with particular attention to the comprehensibility of the content [55]. It is crucial to investigate whether the items apply to Chinese patients and assess their understanding of item wording and expression.

### Structural validity

Structural validity refers to the degree to which a scale accurately reflects the construct dimensions it is intended to measure. HRQOL scales, being based on reflective models, are subject to structural validity evaluation. In this study, all the scales discussed were validated using classical test theory, and factor analysis is the preferred statistical method recommended by guidelines [9]. CFA is used to determine whether the data align with a predefined factor structure of the scale, and studies employing this method are rated as having “very good” methodological quality. In contrast, EFA is utilized for scales without clearly defined latent dimensions, receiving an “adequate” methodological quality rating [12]. In this study, most scales were evaluated using EFA; however, insufficient sample sizes—often fewer than the recommended threshold of five participants per item—resulted in methodological quality downgrades by one or two levels. Two studies assessing the structural validity of the SS-QOL scale [35, 36] applied cluster analysis, a method not aligned with guideline-recommended approaches, leading to an “inadequate” rating for methodological quality. Structural validity studies on the SV-SS-QOL scale [39, 40] produced inconsistent results. Specifically, the two-factor model demonstrated poor fit indices, whereas the one-factor model exhibited better fit. Ted et al. [40] similarly reported poor fit for the two-factor model, suggesting that the scale’s latent factor structure might be unidimensional. For cross-cultural adaptations of established scales, future studies should prioritize CFA to confirm the appropriateness of the latent structure. For the development of new scales, researchers should first employ EFA to identify the dimensional structure, followed by CFA to validate the model fit. Regardless of the method used, ensuring sufficient sample sizes is critical to achieving reliable and valid results.

### Internal consistency

Internal consistency refers to the degree of association among the items within a scale. Since unidimensionality is a prerequisite for explaining internal consistency, structural validity studies should be conducted first to determine the factor structure of the scale before assessing internal consistency [56]. In this review, several studies were rated as “indeterminate” for internal consistency due to the absence of structural validity studies or failure to meet the criterion of “at least low-quality sufficient structural validity.” For instance, the SWAL-QOL scale demonstrated Cronbach’s alpha coefficients above 0.7 for each subdimension. However, as its structural validity was rated as “very low,” the internal consistency was categorized as “indeterminate.” Evaluating internal consistency requires calculating consistency coefficients for each dimension or subscale. For continuous variables, Cronbach’s alpha or Omega coefficients are recommended for each dimension [12]. However, methodological issues were evident in some studies. For instance, in the internal consistency study of the WHOQOL-BREF by Hou et al., item-dimension correlation coefficients were used as statistics, leading to the methodological quality rating of “doubtful”. Similarly, in Li et al.’s study on the QOLI-CAP scale, internal consistency coefficients were reported only for the disease symptom dimension, with no validation of the physiological, psychological, or social dimensions in stroke populations. As a result, the study received an “inadequate” methodological rating. Internal consistency is a key psychometric indicator, directly influencing the grading and recommendation of measurement tools. It is suggested that scholars further investigate the internal consistency of relevant scales to address the research gaps in this field.

### Reliability

The studies included in this research all employed test-retest reliability designs to assess the stability of the scales. Test-retest reliability refers to the consistency of results when the same method is applied to the same subjects over a specified time interval. For quantitative or categorical data, the intraclass correlation coefficient (ICC) or Kappa value is preferred for evaluating test-retest reliability [57]. In this study, the scores of the stroke patients’ HRQOL scales were all quantitative data; thus, the ICC value should have been used to assess test-retest reliability. However, two studies on the SS-QOL scale [35, 37] employed the Kappa value to evaluate the relationship between repeated measurements, which deviates from guideline recommendations, resulting in an “inadequate” rating for methodological quality. The guidelines recommend an appropriate test-retest interval of two weeks; however, some studies opted for intervals shorter than three days. Such short intervals risk introducing recall bias due to the influence of previous measurements, thereby lowering the methodological quality to “inadequate”. Additionally, insufficient sample size is a factor affecting the quality of evidence. In some studies, the sample size was smaller than 100 or even 50, which led to a corresponding reduction in evidence quality by 1 or 2 levels. In the future, it is recommended to improve communication with participants in scale reliability studies to foster cooperation and improve follow-up quality. Moreover, appropriate test-retest intervals should be set based on the progression of the disease, with any deviations from standard intervals justified. Consistency in conditions and environments between measurements must be maintained, and appropriate statistics should be employed to represent test-retest reliability. For the ICC value, researchers should provide detailed descriptions of the calculation models or formulas used to reduce bias and enhance the scientific rigor of the findings.

### Hypotheses testing for construct validity

In this study, the tools used to validate the HRQOL scales for stroke patients included the Barthel Index, Spitzer Quality of Life Index, Fugl-Meyer Assessment of Motor Function, Wechsler Intelligence Scale, SF-36, FCA, Hamilton Rating Scale, NIHSS Scale, Zung Depression Scale, MMSE, SSEQ-C, FAI, NHP, WHOQOL-BREF, SWAL-QOL, and others. These instruments have demonstrated robust reliability and validity in stroke patients or similar populations. However, the methodological quality of the related studies reviewed here was not rated as “very good,” as they reported the correlation coefficients between the scale and comparison scales but did not provide the distribution of measurement data. For example, in the study by He et al. [45], which validated the discriminant validity of the QOL Scale for Stroke, “non-stroke patients” were used as the control group. However, the study failed to specify the exact diseases or conditions within the control group, leading to a methodological quality rating of “doubtful,” and its findings could not substantiate the hypothesis. In future studies involving hypothesis testing of structural validity, it is essential to clearly describe the key characteristics of both groups and provide comprehensive measurement data to minimize bias risk in study design.

*Responsiveness and measurement error*: Responsiveness refers to the ability of the PROM scale to detect changes in the construct over time. In this study, responsiveness was defined as the scale’s capacity to assess changes across various dimensions of quality of life in stroke patients following interventions. Based on a literature review, the research team hypothesized that stroke patients undergoing standardized rehabilitation interventions would show large effect sizes in both total scores and subscale scores of HRQOL. Although specific effect size values were not provided in the paper, they can be calculated using the following formula: Effect size = Mean change (pre- and post-intervention) / Baseline standard deviation (pre-intervention). However, several studies [42, 46]did not report the mean scores of both measurements or the baseline standard deviation, rendering effect size calculations impossible. Consequently, these studies were rated as having “inadequate” methodological quality, and the measurement properties were classified as “indeterminate.”

Measurement error refers to the systematic and random errors in the HRQOL scores of stroke patients that do not accurately reflect true changes in the measured construct [57]. In the study by Ted et al. [40], which examined the measurement error of the SV-SS-QOL scale, a test–retest interval of two months was used. Although this exceeds the guideline-recommended interval of approximately two weeks, the authors justified the interval, noting that the chronic stroke patients in rehabilitation centers exhibited slow recovery, implying stable conditions between measurements. Nevertheless, the study failed to report the minimum important change (MIC), precluding further analysis of the measurement property quality. In future studies on responsiveness and measurement error, it is recommended to provide key data for evaluating attribute quality in detail, to reduce the risk of bias and improve the scientific rigor of findings.

## Conclusion

Two scales—QLICD-ST(SPD) and SAQOL-39 g—met the criteria of “sufficient content validity and at least low-quality sufficient internal consistency,” achieving categories of “A.” These scales are recommended for use in assessing the HRQOL in stroke patients. The remaining 19 scales did not meet the “A” standard and also fell short of the “high-quality evidence of insufficient measurement property” standard, receiving categories of “B.” These scales have some potential for recommendation in the future by refining studies of their measurement properties to improve their applicability. These scales include: WHOQOL-BREF, SF-36, SF-12, Spitzer Quality of Life Index, MHIEC-ST, QLICD-ST(CGD), SIS, Proxy-SIS, SS-QOL, SV-SS-QOL, QOLI-CAP, the TCM Stroke Quality of Life Scale, Cao et al.’s QOL Scale for Stroke Patients, He et al.’s QOL Scale for Stroke Patients, QOLISP, EDQOL, DHI, and SWAL-QOL. Additionally, among the four generic QOL scales reviewed, the SF-36 demonstrated the most comprehensive psychometric evaluation in stroke populations, with superior measurement quality, making it a recommended tool for cross-sectional comparisons of QOL between stroke patients and other populations.

## Limitations

1. Stroke patients often experience psychological and physical issues such as depression and fatigue, which have been identified as key factors contributing to reduced HRQOL. However, this study did not include research on measurement tools assessing these conditions, which may limit the generalizability of our findings to some extent.
2. Due to constraints in human and material resources, this study only included psychometric evaluations of Chinese versions of HRQOL scales for stroke. Future research could build upon this by incorporating psychometric studies of relevant scales from other countries and conducting systematic reviews to identify the optimal HRQOL assessment tool.
3. Finally, although the search strategy was developed in consultation with a medical librarian and included multiple databases with comprehensive search terms, it did not fully incorporate a validated search filter, such as the COSMIN filter or the University of Oxford PROM filter. This may have increased the risk of missing relevant studies and should be acknowledged as a limitation.
